# Lack of Adrenomedullin Aggravates Acute TNBS-Induced Colitis Symptoms in Mice, Especially in Females

**DOI:** 10.3389/fphys.2017.01058

**Published:** 2017-12-19

**Authors:** Sonia Martínez-Herrero, Ignacio M. Larrayoz, Judit Narro-Íñiguez, Susana Rubio-Mediavilla, Alfredo Martínez

**Affiliations:** ^1^Oncology Area, Center for Biomedical Research of La Rioja, Logroño, Spain; ^2^Pathology Service, Hospital San Pedro, Logroño, Spain

**Keywords:** adrenomedullin, inflammatory bowel disease, colitis, 2,4,6-trinitrobenzenesulfonic acid, leucine-rich repeat-containing G-coupled receptor 5, junctional adhesion molecule-A, e cadherin

## Abstract

Adrenomedullin (AM) is a biologically active peptide which has been tested as a new therapy for inflammatory bowel disease (IBD) in animal models and in patients with severe ulcerative colitis. We used an inducible knockout (KO) mouse model for AM to evaluate the effects of endogenous levels of this peptide on the development and degree of pathogenesis of IBD. Acute colitis was induced in mice of both sexes by rectal instillation of 3 mg 2,4,6-trinitrobenzenesulfonic acid (TNBS) in 100 μL of 50% ethanol. Control mice received the same volume of saline in 50% ethanol. During the following 5 days, the weight and the disease severity index of all animals were recorded. After sacrifice, the inflammatory response was macroscopically assessed by analyzing the weight of the colon; by histomorphometrical analysis on histological sections; and by qRT-PCR determination of different inflammatory, adhesion, and regeneration molecules. TNBS administration caused a significantly more severe colitis in KO mice, and especially in females, when compared to wild type (WT) animals. Abrogation of the AM gene caused more severe diarrhea, accompanied by rectal bleeding, anorexia, and a significant increase of colon weight. Histological analysis of TNBS-treated KO mice showed large areas of lymphocyte infiltrates in the mucosa and submucosa, with loss of tissue architecture. No alterations were observed in the expression levels of inflammatory cytokines at the time of sacrifice; meanwhile lack of AM resulted in lower levels of some adhesion molecules and regeneration markers. Taken together, these results support the protective role of endogenous AM against the development of acute colitis, and that its effects are particularly beneficial on females.

## Introduction

Gastrointestinal diseases are attracting renewed interest in biomedicine due to the increased incidence of newly diagnosed cases in recent years (Dahlhamer et al., 2016). Crohn's disease (CD) and ulcerative colitis (UC) are the principal types of inflammatory bowel diseases (IBD), a group of inflammatory conditions characterized by chronic inflammation of the gastrointestinal tract (Leone et al., 2013). Epidemiologic studies revealed that the number of new IBD diagnoses is increasing all around the world (Ponder and Long, 2013; Dahlhamer et al., 2016). Although incidence and prevalence ratios are similar for males and females in IBD, according to data of the “Centers for Disease Control and Prevention” in the United States UC is slightly more common in males, while Crohn's disease is more frequent in women (CDC report, 2015). Furthermore, the prevalence of CD was also higher in females and it increases with age (Lowe et al., 2009; Betteridge et al., 2013). Etiology of IBD remains unclear, however it is viewed as the outcome of a multifactorial process involving an abnormal response of the mucosal immune system to gut microbial communities (Ha et al., 2014); a genetic predisposition (Matricon, 2010; Anderson et al., 2011); and some environmental factors such as pollution (Kaplan et al., 2010), stress (Ponder and Long, 2013), or Western-based diets (Gentschew and Ferguson, 2012). Prior observational studies have suggested a link between exogenous hormone use and risk of IBD (Khalili, 2016). Specifically, some studies have shown an association between oral contraceptive use and risk of CD, and menopausal hormone therapy and risk of UC (Khalili, 2016).

Adrenomedullin (AM) and proadrenomedullin N-terminal 20 peptide (PAMP) are two peptide hormones produced by the same gene, *adm*. They can be found in almost all tissues and body secretions, and exert many physiological functions (Martínez et al., 1997; López and Martínez, 2002). The AM receptor is constituted by two different proteins: a 7-transmembrane domain protein known as calcitonin receptor-like receptor (CLR), and a single transmembrane domain protein called receptor activity modifying protein (RAMP) (Martínez-Herrero and Martínez, 2016). Specific binding sites for AM are also located in many tissues and cell types (López and Martínez, 2002). In the gastrointestinal tract they are especially abundant in the neuroendocrine cells of the mucosa, suggesting a role as a gut hormone for this peptide. Previous studies confirmed that both AM and PAMP regulate many physiological and pathological conditions in the gut (Galanternik et al., 2016). Furthermore, AM and PAMP also act as antimicrobial peptides, constituting a defense mechanism against pathogenic bacteria (Allaker et al., 1999). Recent discoveries, within our laboratory, have confirmed that abrogation of *adm* gene leads to important changes in microbial populations of the gastrointestinal tract under physiological conditions (Martínez-Herrero et al., 2016a). Furthermore, lack of AM/PAMP causes changes in the pattern of expression of the main colon receptor of microorganisms, the toll-like receptor 4 (Martínez-Herrero et al., 2016a).

The therapeutic potential for the administration of AM in gastrointestinal diseases, such as IBD, has been demonstrated in both rodents (Ashizuka et al., 2009) and humans (Ashizuka et al., 2012, 2013a). But the role of endogenous AM in this type of inflammatory pathologies has been scarcely investigated to date (Martínez-Herrero et al., 2016a). To carry out formal studies to deepen our knowledge on the role played by endogenous AM in acute colitis *in vivo*, a knockout (KO) model is needed. Previous attempts to generate a KO for AM, PAMP, both peptides, or the proteins that constitute their receptors, resulted in the dead of all embryos at mid-gestation due to serious vascular anomalies (Shindo et al., 2001; Koyama et al., 2013). To circumvent this problem, we developed an inducible model in which the AM gene can be deleted in adult mice using Cre/loxP technology and a tetracycline-controlled transcriptional activation method (Martínez-Herrero et al., 2016b). These animals survive after *adm* gene abrogation and constitute a suitable model to investigate the role of endogenous AM in intestinal pathophysiology, including IBD.

## Materials and methods

### Ethical permits

All procedures involving animals were carried out in accordance with the European Communities Council Directive (2010/63/UE) and Spanish legislation (RD53/2013) on animal experiments and with approval from the ethical committee on animal welfare of our institution (Órgano Encargado del Bienestar Animal del Centro de Investigación Biomédica de La Rioja, OEBA-CIBIR).

### Generation of inducible knockout mice

Mice where the *adm* gene was surrounded by loxP sequences (“floxed”) were generated in our lab and previously characterized (Fernández et al., 2008). These animals were crossed with transgenic mice expressing Cre recombinase under the control of a tetracycline-responsive promoter element (tetO) (Strain Number 6234, The Jackson Laboratory, Bar Arbor, ME) and with mutant mice having widespread expression of an optimized form of reverse tetracycline-controlled transactivator (rtTAM2) protein (Strain Number 6965, The Jackson Laboratory). All 3 strains had been previously backcrossed to a C57BL/6 genetic background for several generations. For experiments, the following 2 genotypes were selected: normal controls (homozygous for the *adm* WT allele, tetO-Cre, and rtTA) and KO animals (homozygous for the “floxed” *adm* allele, tetO-Cre, and rtTA). All of them were treated with 2 mg/ml doxycycline in the drinking water for 15 days and no experiments were performed until 4 weeks later to allow for microbiota recovery, as described (Martínez-Herrero et al., 2016b).

### Induction of acute colitis with trinitrobenzensulfonic acid

Eighty 16-week old male and female mice were used. Mice were randomly distributed in the following groups: control WT males (WTM) (*n* = 10), TNBS-treated WTM (*n* = 10), control KO males (KOM) (*n* = 10), TNBS-treated KOM (*n* = 10), control WT females (WTF) (*n* = 10), TNBS-treated WTF (*n* = 10), control KO females (KOF) (*n* = 10), and TNBS-treated KOF (*n* = 10). Mice were housed in specific-pathogen free facilities with controlled parameters (temperature 24–25°C, humidity 70–75%, lighting regime 12 h light/12 h darkness) and were fed a regular laboratory diet (Panlab, Barcelona, Spain). The protocol was performed as previously described (Kono et al., 2010), with slight modifications according to Gonzalez-Rey et al. (2006). Briefly, mice were anesthetized with inhaled isoflurane (*IsoFlo*®, Esteve, Barcelona, Spain) following a 6-h fast. To induce colitis, 3 mg 2,4,6-trinitrobenzenesulfonic acid (TNBS, Sigma Aldrich, St Louis, MO) dissolved in 0.1 ml of 50% ethanol were instilled into the colon using a 3.5 F polyurethane catheter, previously impregnated in vaseline, connected to a 0.3cc syringe without a needle. The catheter was introduced till the end of the colon, which was about 3 cm in males and 2.5 cm in females. Control mice received the same volume of saline in 50% ethanol. Following the instillation, the animals were maintained in a head-down position for 1 min to prevent leakage of the intracolonic instillate. Animals were sacrificed using an anesthetic (*Xylagesic*®, Laboratorios Calier, S.A., Barcelona, Spain + *Imalgene*®, Merial, Lyon, France) overdose administered intraperitoneally 5 days after TNBS instillation. No mice died during the 5 days of the experiment. If particular animals presented dehydration symptoms, they received rehydration therapy (*Ringer's Lactate Solution*®, Braun, Barcelona, Spain). Fluid homeostasis was evaluated by lifting gently the skin on the animal's back. If skin turgor is reduced and forms tent-like wrinkles, this was considered as a symptom of clinical dehydration (NAVC Conference, 2005). The rehydration therapy consists in an intraperitoneal injection of Ringer's Lactate Solution replacing 20% of the total blood volume of the animal (NAVC Conference, 2005). Total blood volume is estimated as 6% of the total body weight. Subsequent subcutaneous administrations of the same solution were provided to dehydrated mice. The total volume to be administered each day was calculated following The Federation of Law Societies of Canada's guidelines.

### Clinical assessment of acute colitis

Mice were weighed and observed daily. Clinical colitis symptoms were blindly scored according to the system described by Gommeaux et al. (2007) considering parameters such as inactivity, behavior, percent weight loss relative to baseline, diarrhea, prolapse, and rectal bleeding. Final score was used as a surrogate of colitis severity.

### Tissue harvesting and macroscopical assessment of colonic damage

Tissue harvesting was performed as previously described (Martínez-Herrero et al., 2016a). Briefly, the entire colon was dissected and rinsed with ice-cold phosphate buffer solution (PBS) to remove fecal residues. Then, the last 5 cm of colon (measured from the anus) were weighed. Photographs of colon samples were taken using an *EOS 50D* camera (Canon, Tokyo, Japan). Central portions of colonic tissue were fixed in 10% buffered formalin, processed for paraffin embedding, and sections were stained with hematoxylin and eosin (H&E). Distal and proximal sections of the colon (2 cm each) were snap-frozen in liquid nitrogen and stored at −80°C for further analysis.

### Histopathological studies of the colon

Three non-consecutive H&E-stained slides from each colon sample were used for histological evaluation of colonic damage. Slides were coded to prevent observer bias during evaluation. All sections were examined in an Eclipse 50i microscope (Nikon, Amsterdam, Netherlands). The height of the mucosa, the submucosa, and the intestinal wall were used as a surrogate measure of inflammation. The measurements were always performed by computerized morphometry, as previously described (Barajas-Espinosa et al., 2011; Mello et al., 2012). Briefly, the measurements of the thickness of the colonic layers were always performed in six random fields, selecting sites where at least five contiguous and intact crypts were present. The captured image was digitized, transferred, and analyzed using FIJI software. The program automatically provided the average of measured values with the standard error of each segment. In addition, a pathologist examined the slides blindly and tissues were scored using the histological colitis scoring method described by Hayashi et al. (2011). This score tests for three tissue characteristics; inflammation severity: grade 0 = none, grade 1 = mild, grade 2 = moderate, and grade 3 = severe; crypt damage: grade 0 = none, grade 1 = basal 1/3 damaged, grade 2 = basal 2/3 damaged, grade 3 = crypts lost and surface epithelium present, and grade 4 = crypts and epithelium lost; and percent involvement: grade 0 = 0%, grade 1 = 1–25%, grade 2 = 26–50%, grade 3 = 51–75%, and grade 4 = 76–100%. The inflammation score is calculated as the sum of the inflammation severity multiplied by the percent involvement (maximum score 12) and the crypt damage multiplied by the percent involvement (maximum score 16). The total maximum score was 28.

### RNA isolation and quantitative real-time PCR

RNA isolation, cDNA synthesis, and qRT-PCR were performed as previously described (Larráyoz et al., 2012a). Briefly, total RNA was isolated from full thickness distal colon fragments using Qiagen RNAseasy Mini Kit with DNAse digestion step performed (Qiagen, Hilden, Germany) according to manufacturer's instructions. Total RNA (1 μg) of each sample was reverse transcribed using the SuperScript® III Reverse Transcriptase Kit (Thermo Fisher Scientific). The synthesized cDNA was amplified by qRT-PCR with a 7300 real-time PCR System (Applied Biosystems, Foster City, CA, USA). Cycling conditions were an initial denaturation at 95°C for 10 min, followed by 40 cycles of 95°C for 15 s and 60°C for 1 min. At the end, a dissociation curve was implemented from 60 to 95°C to validate amplicon specificity (Rey-Funes et al., 2016). Gene expression was calculated using relative quantification by interpolation into a standard curve. As recommended by Eissa et al. (2017), three different housekeeping genes (GAPDH, Tbp, and Eef2) were used for normalization. NormFinder program was used for calculating the expression stability of selected reference genes (Eissa et al., 2017) and specific housekeeping genes were selected for each experimental group. Relative gene expression levels were quantified using RQ software (Applied Biosystems). Target genes and primers are described in Table 1.

**Table 1:** Primer sequences used for quantitative real time PCR measurements.

**Gene**	**Primer**	**Sequence (5′ → 3′)**	**Expected Amplicon Size**
GAPDH	Sense	CAT GGC CTT CCG TGT TCC TA	55 bp
	Antisense	GCG GCA CGT CAG ATC CA	
TBP	Sense	ACC GTG AAT CTT GGC TGT AAA C	86 bp
	Antisense	GCA GCA AAT CGC TTG GGA TTA	
Eef2	Sense	TGT CAG TCA TCG CCC ATG TG	123 bp
	Antisense	CAT CCT TGC GAG TGT CAG TGA	
Adrenomedullin	Sense	ATT GAA CAG TCG GGC GAG TA	130 bp
	Antisense	CTT GGTCTT GGG TTC CTC TG	
TNF-α	Sense	GCA CCA CCA TCA AGG ACT CA	51 bp
	Antisense	TCG AGG CTC CAG TGA ATT CG	
IL-1β	Sense	ACA CTC CTT AGT CCT CGG CCA	51 bp
	Antisense	TGG TTT CTT GTG ACC CTG AGC	
IL-6	Sense	ATG GAT GCT ACC AAA CTG GAT	139 bp
	Antisense	TGA AGG ACT CTG GCT TTG TCT	
ZO-1	Sense	GGA GCA GGC TTT GGA GGA G	163 bp
	Antisense	TGG GAC AAA AGT CCG GGA AG	
Occludin	Sense	GTC CTC CTG GCT CAG TTG AA	165 bp
	Antisense	CGG ACA TGG CTG ATG TCA CT	
JAM-A	Sense	AAC CCA TGG CTG ATT CCC AG	201 bp
	Antisense	TAG AGG ACG ACT TGG GGA GG	
B-catenin	Sense	TGT CCT GTG AAG CCC GC	225 bp
	Antisense	GCT TTT CTG TCC GGC TCC AT	
E-cadherin	Sense	GGA CAG CAA CAT CAG CGA AC	190 bp
	Antisense	GCT ACC ATC AAG AGC AGG CA	
Desmoglein-2	Sense	CCT CTT GCC ATT GAC CGA CT	56 bp
	Antisense	AAT GGC TGG GGT TCT GTG AG	
Connexin 26	Sense	GCT TAG TCG CTT AGT CGG CA	120 bp
	Antisense	CAC GGA GGC TTC TGG AGT TT	
B-actin	Sense	CCT AAG AGG AGG ATG GTC GC	230 bp
	Antisense	CTC AAC ACC TCA ACC CCC TC	
LGR5	Sense	CCT ACT CGA AGA CTT ACC CAG T	165 bp
	Antisense	GCA TTG GGG TGA ATG ATA GCA	
Wnt5a	Sense	CAA CTG GCA GGA CTT TCT CAA	121 bp
	Antisense	CAT CTC CGA TGC CGG AAC T	
Egfr	Sense	GCC ATC TGG GCC AAA GAT ACC	101 bp
	Antisense	GTC TTC GCA TGA ATA GGC CAA T	
ERBB2	Sense	GCT AGA GCG GCT TCT GAG AAA	111 bp
	Antisense	ACC ACA GGG TCT ACC ACT TCC	

*The annealing temperature was 60°C for all primers*.

### Statistical analysis

All data sets were analyzed for normalcy and homoscedasticity. Normal data were analyzed by Unpaired Student's *t*-test, by 1-way ANOVA, or by 2-way ANOVA followed by Tukey's Multiple Comparison test (MCT). Data that did not follow a normal distribution were compared by Kruskal-Wallis test followed by Dunn's *Post-hoc* test. A *p*-value < 0.05 was considered statistically significant. All these studies were performed with GraphPad Prism version 5.02 (GraphPad Software, Inc. La Jolla, CA).

## Results

**Figure 1:**
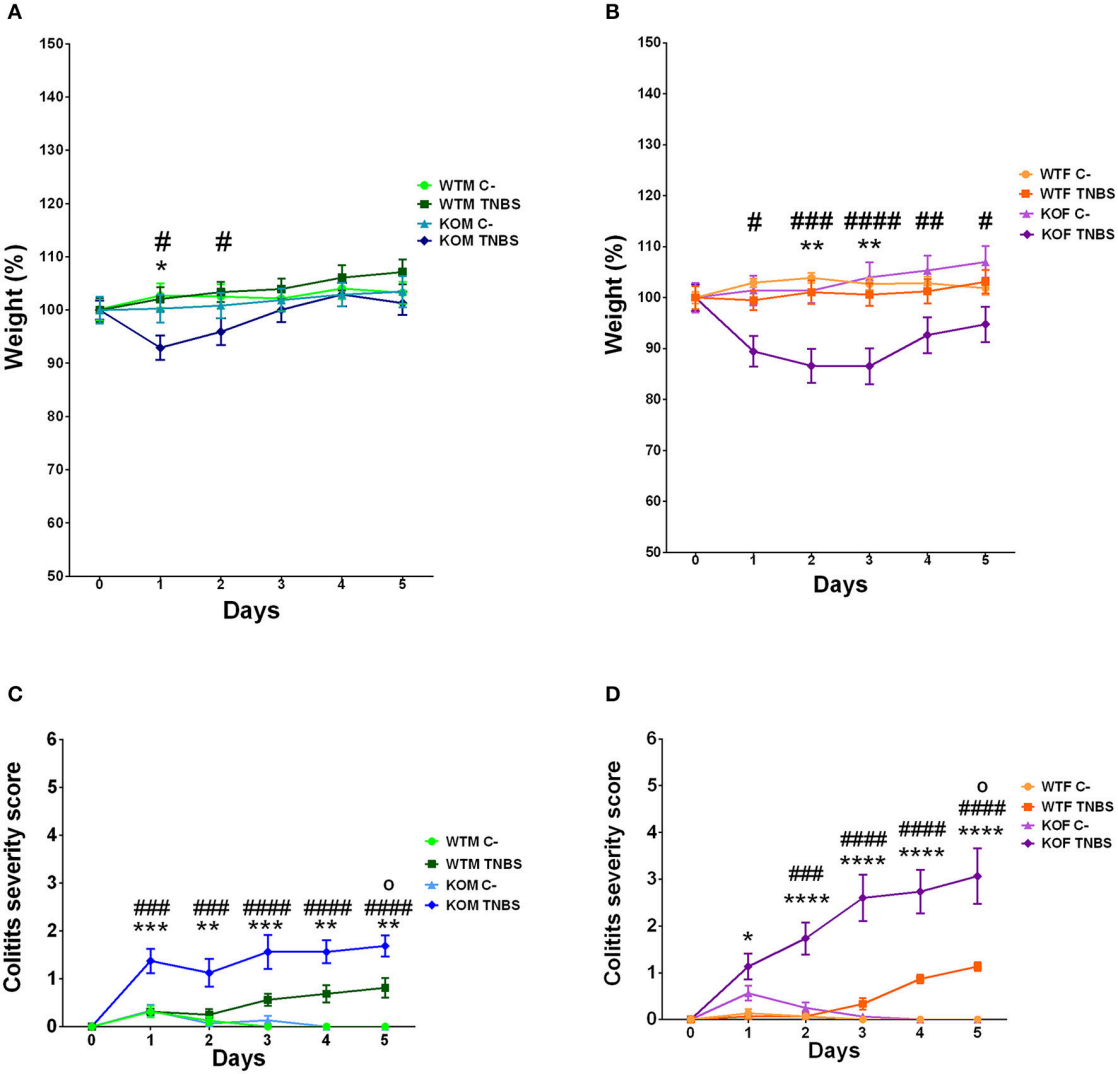
Body weight evolution and clinical assessment of colitis in mice during the 5 days of procedure. Males **(A)** and females **(B)** were weighed daily. Abrogation of AM results in a significant weight loss among TNBS-treated mice, which is higher among females. Colitis symptoms were evaluated on a 0–12 point scale in males **(C)** and females **(D)**. TNBS-treated KO animals showed more severe disease symptoms reaching higher final scores than their treated WT littermates. **(A–D)** Data are shown as mean ± SEM. **(A–D)** Two way ANOVA + Tukey's MCT. Asterisks represent statistically significant differences between TNBS-treated KO versus TNBS-treated WT mice; ^*^p < 0.05. ^**^p < 0.01. ^***^p < 0.001. ^****^p < 0.0001. Pound signs represent statistically significant differences between TNBS-treated KO vs. vehicle-treated KO mice; ^#^*p* < 0.05. ^##^*p* < 0.01. ^###^*p* < 0.001. ^####^*p* < 0.0001. Symbol ° represents statistically significant differences between TNBS-treated WT vs. vehicle-treated WT mice; °*p* < 0.05. WTM, male wild type mice; WTF, female wild type mice; KOM, male knockout mice; KOF, female knockout mice.

**Figure 2:**
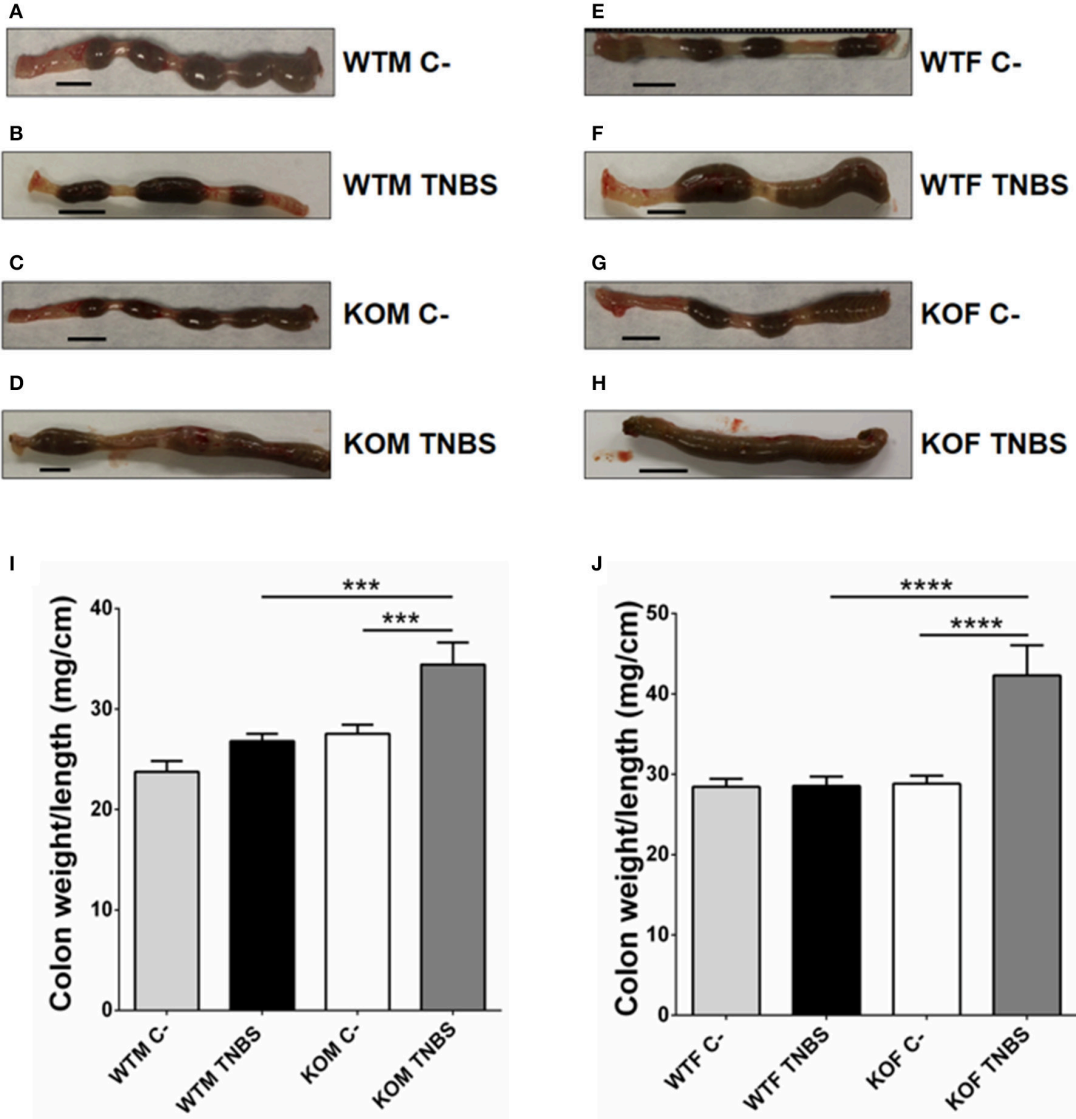
Macroscopic evidence of inflammation in TNBS-treated mice. Photographs of the last 5 cm of the colon (form the anus) from the 4 experimental groups in male **(A–D)** and female **(E–H)** mice, and relative weight of the colon **(I,J)** of the same animals. TNBS treatment did not alter the external aspect of the colon in the treated WTM group **(B)** but it caused local inflammation on the colon in KOM and the presence of diarrhea can be appreciated **(D)**. In females, TNBS treatment caused clinical symptoms in WTF and their colons appeared more inflamed and the feces were semi-solid **(F)** when compared with feces of untreated **(C-)** WTF. KOF instilled with TNBS exhibited a more severe inflammation and the presence of diarrhea and bloody stools can be appreciated **(H)**. A significant augmentation in the weight/length ratio of the colon can be observed in TNBS-treated KOM and KOF mice when compared with their respective controls and treated WT counterparts. **(I,J)** Data are shown as mean ± SEM. **(I,J)** One way ANOVA + Tukey's MCT. ^***^p < 0.001; ^****^p < 0.0001. Same groups as in Figure 1. Scale bar = 5 mm.

We have presented the differences experienced by WT and KO animals after TNBS treatment in body weight (Figures 1A,B), disease severity scores (Figures 1C,D), colonic weight/length ratio (Figures 2I,J), mucosa and submucosa thickness (**Figures 4E,F**, **5E,F**), and histopathological scores (**Figures 4G**, **5G**) separately from the differences experienced between treated males and females of the same genotype (**Figure 6**).

### Lack of endogenous adrenomedullin accelerates and aggravates acute colitis symptoms

WT mice of both sexes treated with TNBS maintained body weight (Figures 1A,B; two way ANOVA; Tukey's MCT, *p* > 0.05 TNBS-WTM vs. Sham-WTM; *p* > 0.05 TNBS-WTF vs. Sham-WTF) and only exhibited mild colitis symptoms when compared with their vehicle-treated WT littermates at day five (Figures 1C,D; two way ANOVA; Tukey's MCT, *p* < 0.05 TNBS-WTM vs. Sham-WTM; *p* < 0.05 TNBS-WTF vs. Sham-WTF). No differences according to sex were observed among treated WT animals (**Figure 6C**, two way ANOVA; Tukey's MCT, *p* > 0.05 TNBS-WTM vs. TNBS-WTF). KOM mice treated with TNBS experienced a slight weight loss during the 2 days following rectal instillation when compared with their control vehicle-treated counterparts (Figure 1A, two way ANOVA; Tukey's MCT, *p* < 0.05), which paralleled the occurrence of harsher colitis symptoms (Figure 1C, two way ANOVA; Tukey's MCT, *p* < 0.01). After the administration of rehydration therapy they recovered their initial weight. KOF instilled with TNBS suffered a more profound and sustained weight loss (larger than 20% in a quarter of the sample; Figure 1B, two way ANOVA; Tukey's MCT, *p* < 0.05 TNBS-KOF vs. Sham-KOF at day one, *p* < 0.01 TNBS-KOF vs. TNBS-WTF at days two and three). TNBS-treated KOF also presented more serious symptoms of colitis (Figure 1D, two way ANOVA; Tukey's MCT, *p* < 0.001 TNBS-KOF vs. Sham-KOF at day two; *p* < 0.0001 TNBS-KOF vs. TNBS-WTF from day three to five). Furthermore, TNBS-treated KOF experienced worse colitis symptoms than their treated male littermates (**Figure 6C**, two way ANOVA; Tukey's MCT, *p* < 0.05 TNBS-KOM vs. TNBS-KOF at day four, *p* < 0.01 TNBS-KOM vs. TNBS-KOF at day five).

The macroscopic inspection of colon and rectum tissues provided evidence of different degrees of colonic inflammation in null-mice (Figure 2). In TNBS-treated KO mice (Figures 2D,H), the presence of liquid and bloody stools, and more hemorrhagic areas, were clearly different from their TNBS-treated WT littermates (Figures 2B,F) whose colons maintained a more similar aspect to those of control animals of both genotypes (Figures 2A,C,E,G). TNBS treatment did not cause any variation in the colon weight/length ratio in WT mice (Figure 2I, one way ANOVA; Tukey's MCT, *p* > 0.05 TNBS-WTM vs. Sham-WTM). However, in TNBS-treated KOM a significant increase in relative colon weight was observed when compared with their untreated KO controls and with treated WTM (Figure 2I, one way ANOVA; Tukey's MCT, *p* < 0.001 TNBS-WTM vs. TNBS-KOM; *p* < 0.001 TNBS-KOM vs. Sham-KOM). This increase was even greater in the TNBS-treated KOF group (Figure 2J, one way ANOVA; Tukey's MCT, *p* < 0.0001 TNBS-WTF vs. TNBS-KOF; *p* < 0.001 TNBS-KOF vs. Sham-KOF) even when compared with treated null-males (**Figure 6D**, one way ANOVA; Tukey's MCT, *p* < 0.01 TNBS-KOF vs. TNBS-KOM).

### Abrogation of the *Adm* gene increases histological colonic damage in TNBS treated mice

**Figure 3:**
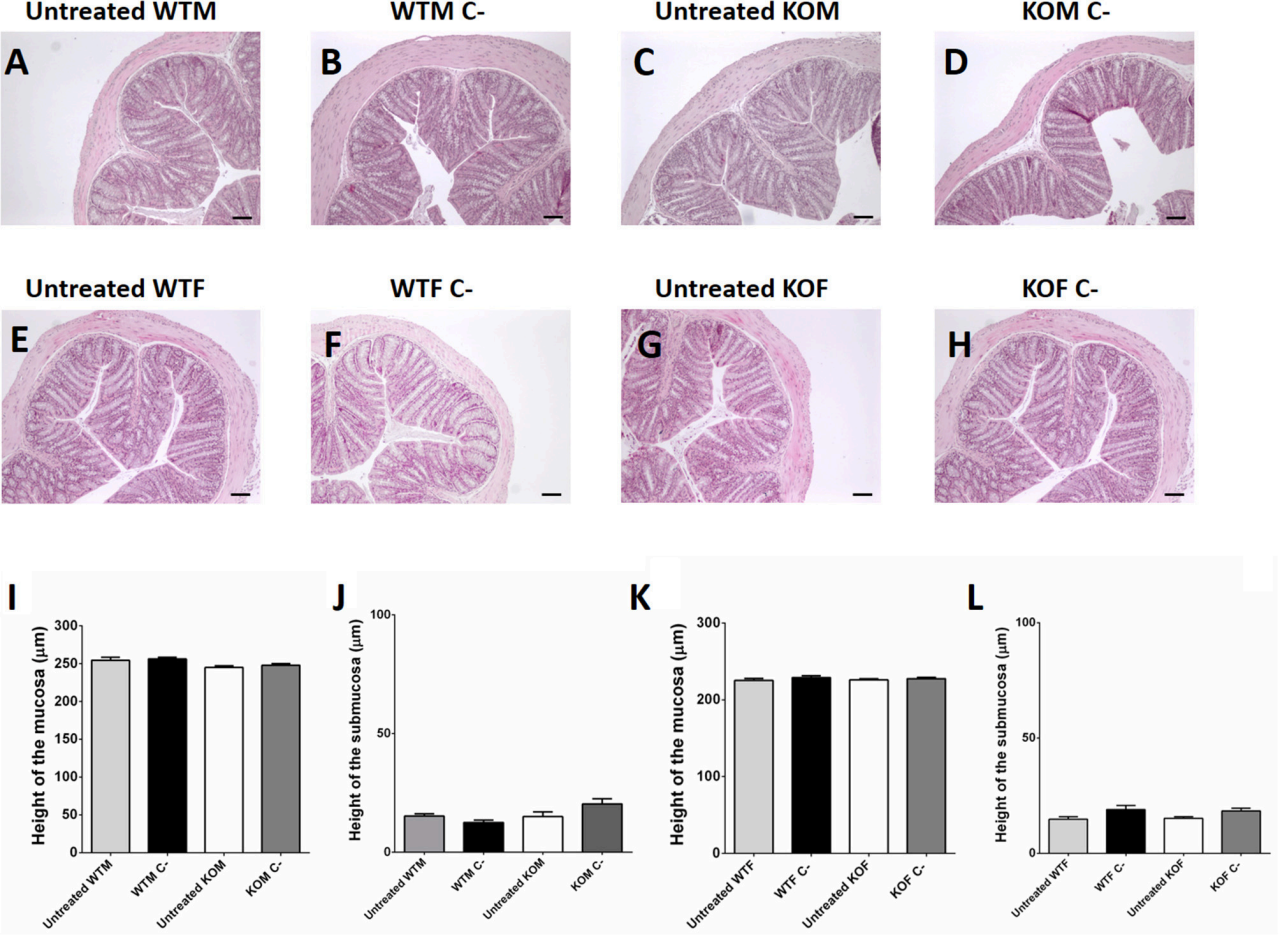
Microscopic evaluation of Ethanol-instillation action in the colon. Some preliminary experiments were performed to investigate whether control treatment with 50% ethanol in NaCl (C-) had any impact in tissue architecture when compared with colonic sections of animals which were not instilled (Untreated). Histological analysis of paraffin sections of males **(A–D)** and females **(E–H)** of both genotypes revealed that 50% ethanol in saline instillation did not cause any significant alteration in the morphology of the colon. Morphometric analysis revealed that control treatment with ethanol did not modify the height of the colonic wall neither of the mucosa **(I,K)** nor of the submucosa **(J,L)**. Data are shown as mean ± SEM. **(I,J)** One way ANOVA + Tukey's MCT. Same groups as in Figure 1. Five animals per group. Scale bar = 50 μm.

**Figure 4:**
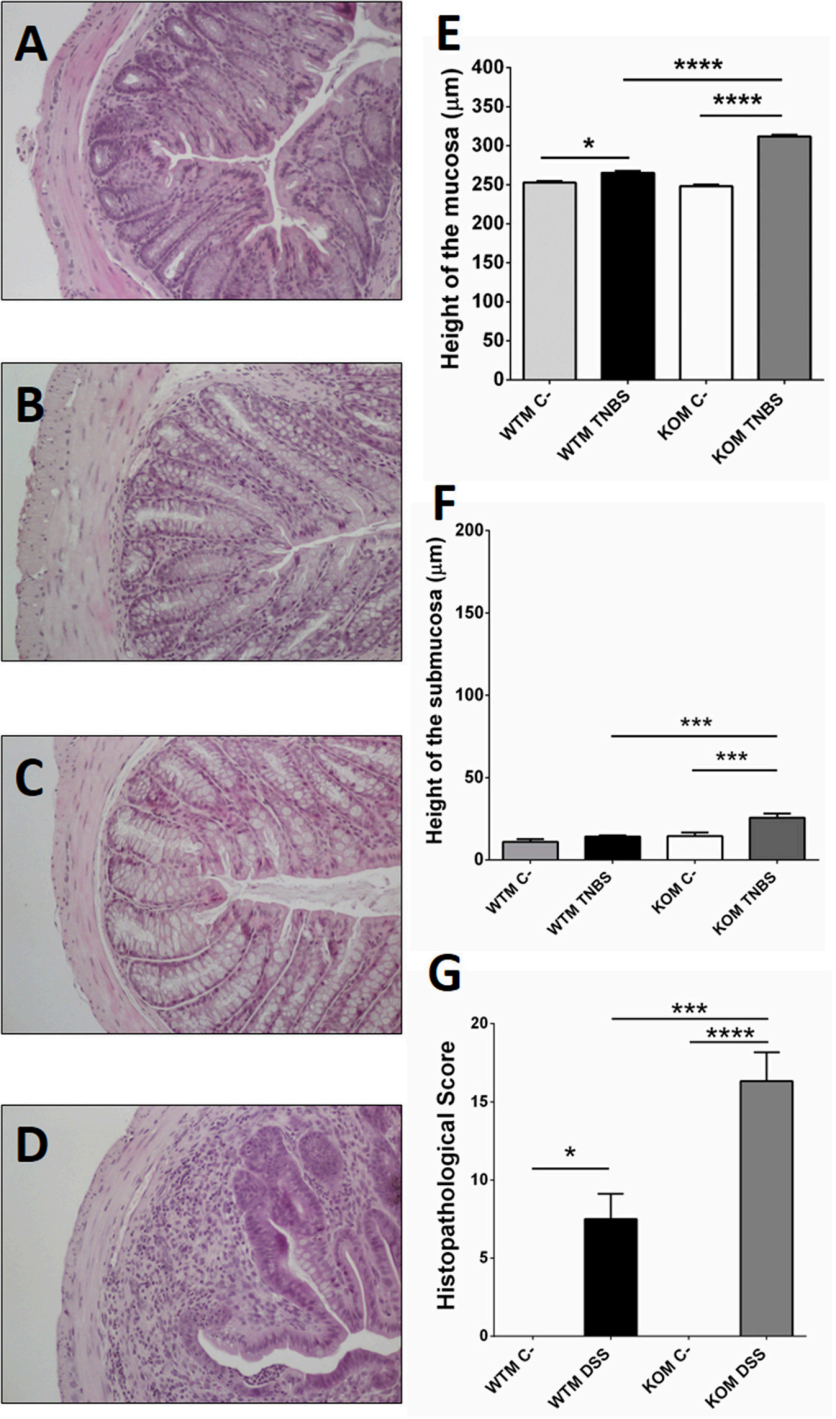
Microscopic evaluation of TNBS-induced damage in males. Histological analysis of paraffin sections of WT **(A,B)** and KO **(C,D)** mice treated with vehicle **(A,C)** or with TNBS **(B,D)**. TNBS treatment did not cause any significant change in microarchitecture of the colon in treated WTM **(B)** when compared with their sham littermates **(A)**. A higher level of inflammation, inflammatory infiltration, and loss of tissue architecture is observed in TNBS-treated KOMs **(D)** when compared to the other experimental groups. Morphometric analysis revealed that TNBS treatment increased the height of the mucosa layer of both genotypes **(E)**, being this thickening greater in treated null-males. No change was observed at the colonic submucosa of TNBS-WTM; meanwhile in KOM inflammation of the intestinal wall was extensive to the submucosa layer **(F)**. The histopathological score was significantly higher after TNBS treatment in both genotypes, but again this result was much more significant in the absence of AM **(G)**. **(E–G)** Data are shown as mean ± SEM. **(E–G)** One way ANOVA + Tukey's MCT. ^*^p < 0.05; ^***^p < 0.001; ^****^p < 0.0001. Same groups as in Figure 1. Scale bar = 100 μm.

**Figure 5:**
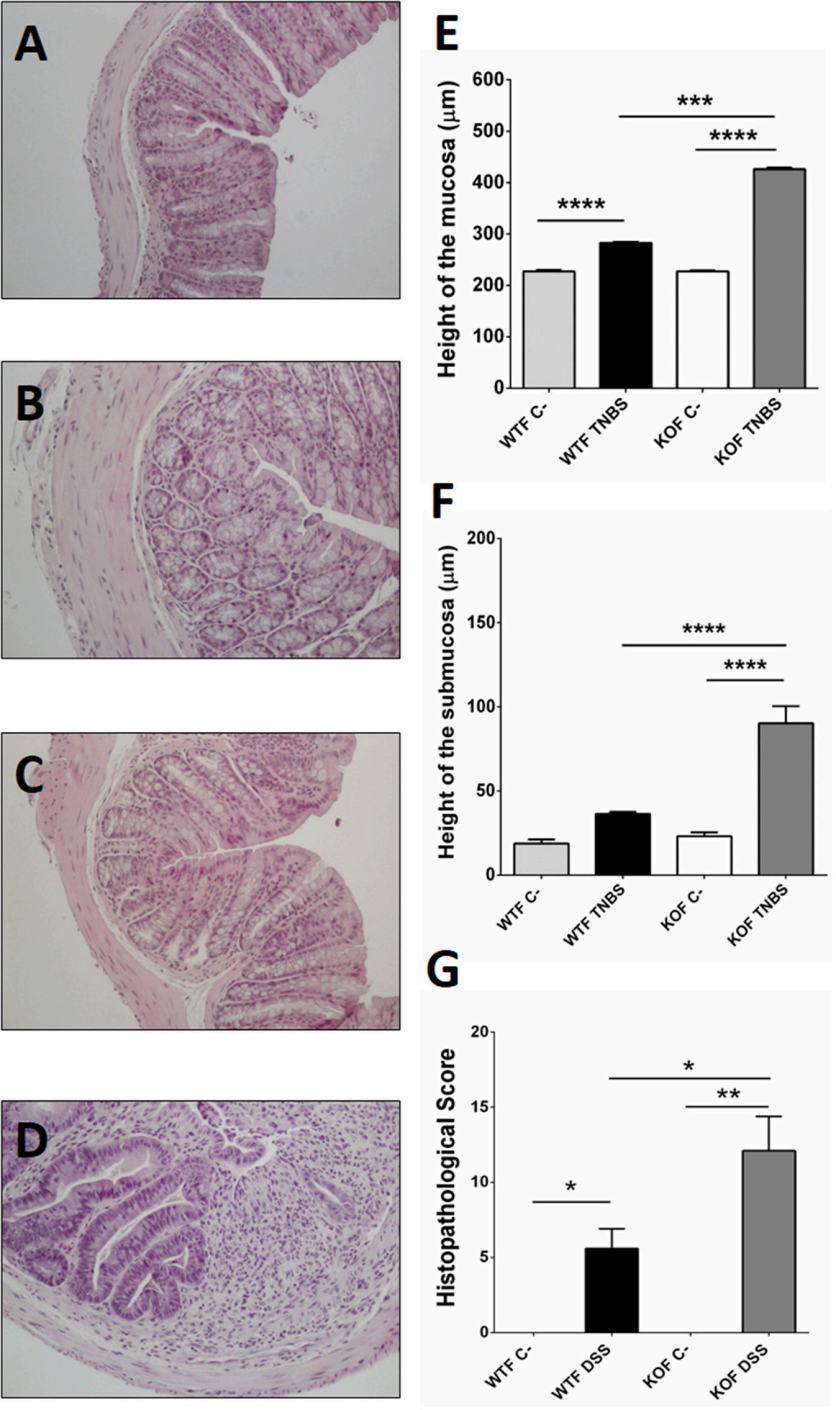
Microscopic evaluation of TNBS-induced damage in females. Histological analysis of paraffin sections of WT **(A,B)** and KO **(C,D)** mice treated with vehicle **(A,C)** or with TNBS **(B,D)**. TNBS treatment did not cause major changes in colonic organization or architecture among WTF **(B)** when compared with untreated WTF, however some inflammatory cells could be observed dispersed through the mucosa. Inflammation, extensive inflammatory infiltration, and loss of tissue architecture were evident in TNBS-treated KOFs **(D)** when compared to the other experimental groups. Histomorphometrical analyses revealed that in female groups, treatment with TNBS caused inflammation of the colonic mucosa in WT and KO **(E)**, being more severe among the KOs. However, it only triggered an inflammatory response in the submucosa of null-female mice **(F)**. As expected, the histopathological score was significantly higher after TNBS treatment, but that response was more elevated in the absence of AM **(G)**. **(E–G)** Data are shown as mean ± SEM. **(E–G)** One way ANOVA + Tukey's MCT. ^*^p < 0.05; ^**^p < 0.01; ^***^p < 0.001; ^****^p < 0.0001. Same groups as in Figure 1. Scale bar = 100 μm.

Preliminary experiments showed that rectal instillation of saline in 50% ethanol did not cause any significant alteration in the colonic architecture of the gut (Figures 3, 4A,C, 5A,C). Despite the absence of macroscopical inflammation signs among WT animals, inspection of histological colonic sections of TNBS-treated WT mice showed the presence of small isolated areas with inflammatory cells, mainly in the mucosa, being more abundant among females, while most of the tissue maintained its typical micro architecture (Figures 4B, 5B). In TNBS-treated KO animals a more abundant inflammatory cell infiltration, which caused a transmural inflammation involving all layers of the colon wall, and loss of epithelial cells was observed (Figures 4D, 5D).

**Figure 6:**
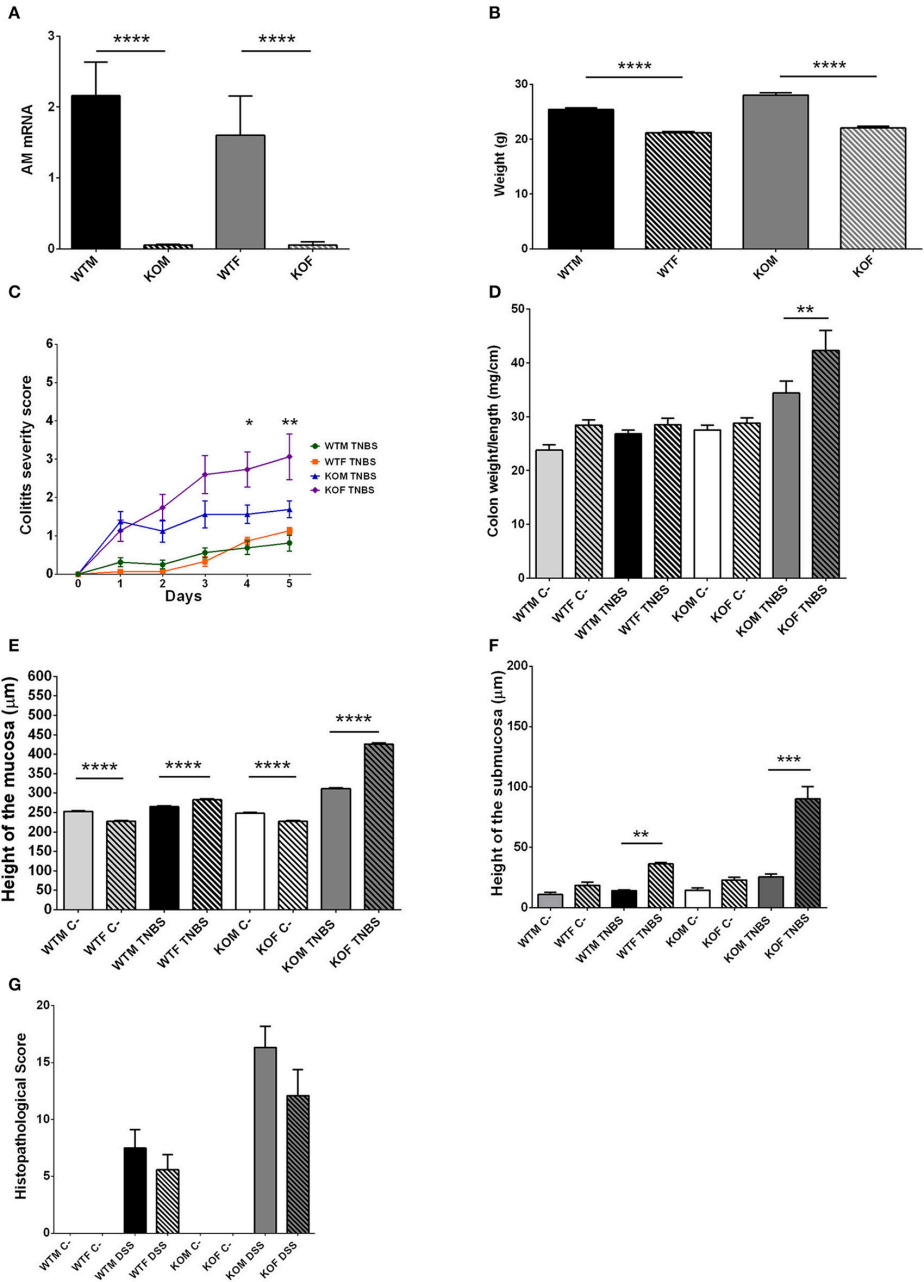
Sexual differences observed in TNBS-induced colitis. All results point to a higher degree of pathogenesis among female groups of both genotypes, being even more severe in KO females. To investigate the reasons for these differences, we first checked that abrogation of *adm* gene was completely achieved in both sexes **(A)**. Another possibility was that the diverse susceptibilities may be due to differences in the weight of males versus females at the beginning of the experiment **(B)**. TNBS treatment did not cause differences in clinical symptoms **(C)** or local inflammation **(D)** between WT males and females. However, lack of AM provokes a significant worsening **(C)** and more severe inflammation of the colon **(D)** in treated-KOF when compared with treated null-males. Morphometric studies of colonic sections revealed that in normal conditions (sham-groups) males had a thicker mucosa **(E)** than females, but no significant sexual differences could be observed in the submucosa layer **(F)**. TNBS instillation reversed these conditions, thus treated females presented a thicker mucosa **(E)** and submucosa **(F)** than treated males. However, no sexual differences were observed when comparing the histological score obtained by treated males and treated females **(G)**. **(A–G)** Data are shown as mean ± SEM. **(A,B,D–G)** One way ANOVA + Tukey's MCT. **(C)** Two way ANOVA + Tukey's MCT. ^*^p < 0.05; ^**^p < 0.01; ^***^p < 0.001; ^****^p < 0.0001. Same groups as in Figure 1.

Histomorphometrical analysis revealed that saline in 50% ethanol instillation did not modify the thickness of colonic layers between genotypes (Figures 4E,F, 5E,F; one way ANOVA, Tukey's MCT, *p* > 0.05 for Sham-WTM vs. Sham-KOM; and Sham-WTF vs. Sham-KOF). In control conditions, males presented a thicker intestinal mucosa (Figure 5E, one way ANOVA; Tukey's MCT, *p* < 0.0001 Sham-WTM vs. Sham-WTF; *p* < 0.0001 Sham-KOM vs. Sham-KOF). TNBS treatment caused a significant increase in the thickness of the mucosa layer among treated WT animals when compared with their control littermates (Figure 4E, one way ANOVA; Tukey's MCT, *p* < 0.05 in males. Figure 5E, one way ANOVA; Tukey's MCT, *p* < 0.0001 in females), being this increase higher in TNBS-WTF when compared with TNBS-WTM, thus reversing the normal situation observed in sham animals (Figure 6E, one way ANOVA; Tukey's MCT, *p* < 0.0001). No changes were observed in the submucosa of treated WT mice (Figures 4F, 5F; one way ANOVA, Tukey's MCT, *p* > 0.05). There was also a significant difference in thickness of the mucosa layer of null-mice after TNBS treatment when compared with untreated null-mice (Figure 4E, one way ANOVA; Tukey's MCT, *p* < 0.0001 in males. Figure 5E, one way ANOVA; Tukey's MCT, *p* < 0.0001 in females) and this thickening was greater than that observed in WT animals treated with TNBS (Figure 4E, one way ANOVA; Tukey's MCT, *p* < 0.0001 TNBS-WTM vs. TNBS-KOM) (Figure 5E, one way ANOVA; Tukey's MCT, *p* < 0.001 TNBS-WTF vs. TNBS-KOF). Among KO animals, inflammation was again much more severe in KOF than in KOM (Figure 6E, one way ANOVA; Tukey's MCT, *p* < 0.0001 TNBS-KOM vs. TNBS-KOF); and the thickening of the intestinal wall was extensive to the submucosa in null-animals (Figure 4F; one way ANOVA; Tukey's MCT, *p* < 0.001 TNBS-WTM vs. TNBS-KOM; *p* < 0.001 TNBS-KOM vs. Sham-KOM) (Figure 5F; one way ANOVA; Tukey's MCT, *p* < 0.0001 TNBS-WTF vs. TNBS-KOF; *p* < 0.0001 TNBS-KOF vs. Sham-KOF). The inflammation of the colonic submucosal layer was significantly higher in treated females of both genotypes when compared with treated males (Figure 6F; one way ANOVA; Tukey's MCT, *p* < 0.01 TNBS-WTF vs. TNBS-WTM; *p* < 0.001 TNBS-KOF vs. TNBS-KOM).

Inflammation, as measured by the histopathological score, was significantly higher in treated WT groups (Figure 4G; one way ANOVA; Tukey's MCT, *p* < 0.05 TNBS-WTM vs. Sham-WTM; Figure 5G; one way ANOVA; Tukey's MCT, *p* < 0.05 TNBS-WTF vs. Sham-WTF). The score obtained by TNBS-treated KO animals showed a more severe inflammation in null-mice when compared with their sham and WT counterparts (Figure 4G; one way ANOVA; Tukey's MCT; *p* < 0.0001 in TNBS-WTM vs. TNBS-KOM; *p* < 0.001 in TNBS-KOM vs. Sham-KOM; Figure 5G; one way ANOVA; Tukey's MCT; *p* < 0.05 in TNBS-WTF vs. TNBS-KOF; *p* < 0.01 in TNBS-KOF vs. Sham-KOF). Only differences between genotypes but not between sexes were observed when comparing the scores assigned to all TNBS-treated groups (Figure 6G, one way ANOVA, Tukey's MCT; *p* > 0.05).

### No changes were observed in the expression levels of inflammatory cytokines at the time of sacrifice

**Figure 7:**
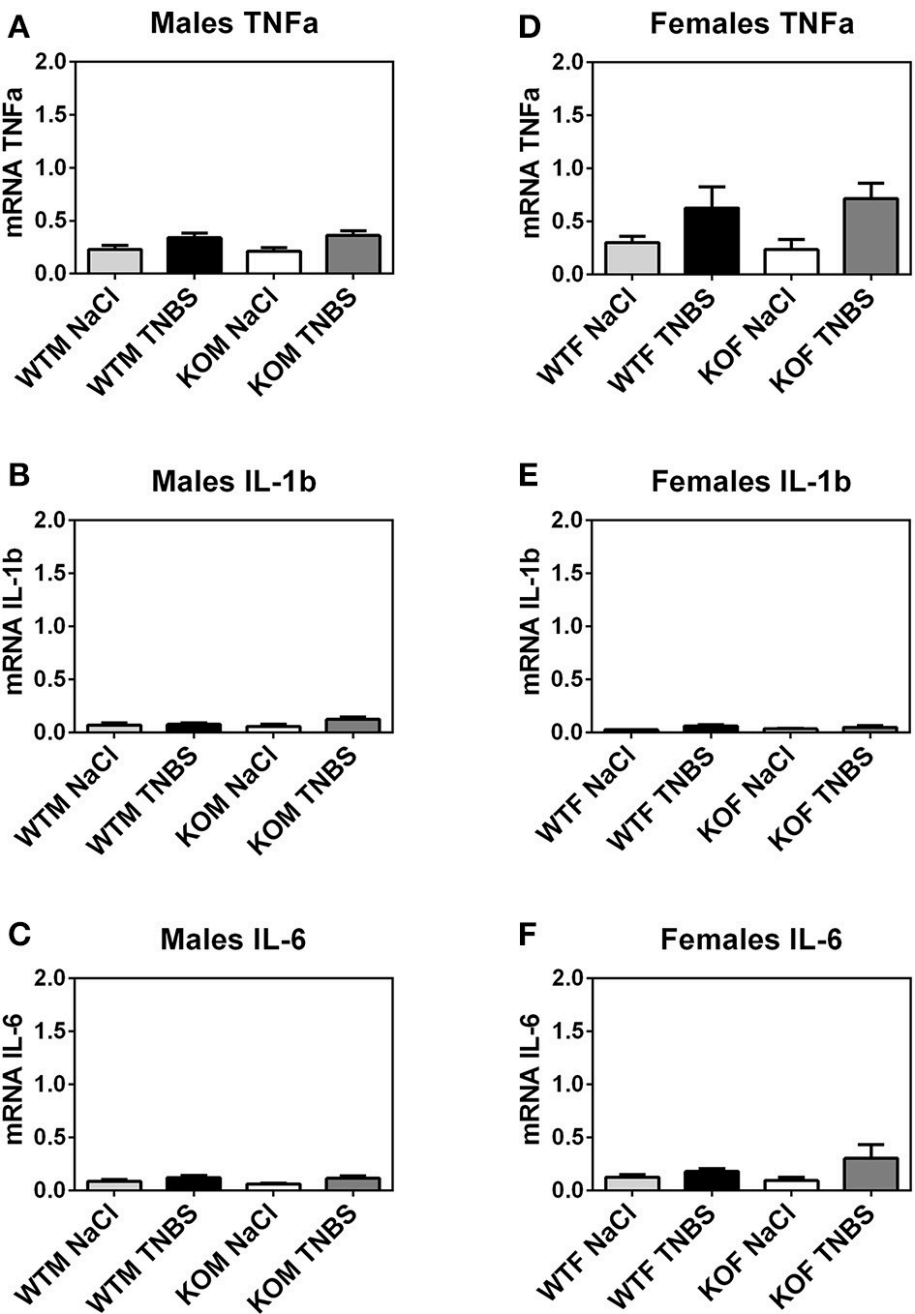
Lack of AM did not modify cytokine levels at the colon in TNBS-induced colitis. mRNA expression of the principal pro-inflammatory molecules was evaluated by qRT-PCR in the colon of male **(A-C)** and female **(D-F)** animals. Inflammatory markers include TNFα **(A,D)**, IL-1β **(B,E)**, and IL-6 **(C,F)**. No significant variation was observed among the studied groups. Data are shown as mean ± SEM. Kruskal Wallis + Dunn's *Post-hoc* test. Same groups as in Figure 1.

**Figure 8:**
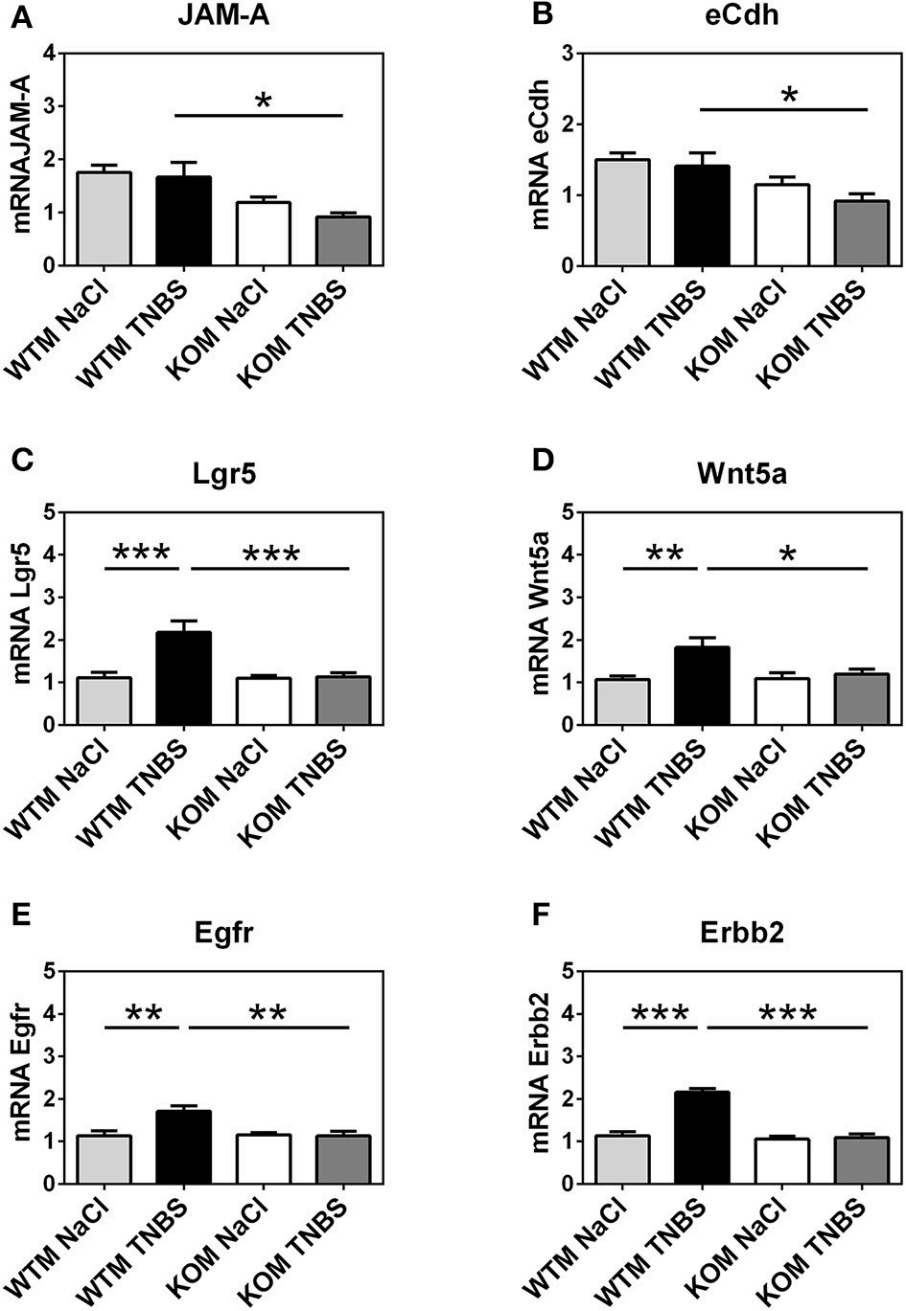
Effect of endogenous AM on epithelial cellular adhesions and regenerative responses during acute episode of colitis caused by rectal instillation of TNBS in males. Expression of 2 adhesion molecules and 4 regenerative biomarkers were evaluated by qRT-PCR in colon samples using GAPDH as housekeeping gene. Presence of *adm* gene prevented from a significant decrease in the expression of JAM-A **(A)** and eCdh **(B)** after TNBS instillation when treated-WTM are compared with their respective null-littermates. Rectal instillation of TNBS caused a significant increase of the 4 regenerative molecules studied **(C–F)** in WTM. Interestingly, this overexpression was not observed in the KO animals. **(A–F)** Data are shown as mean ± SEM. **(A–E)** Two way ANOVA + Tukey's MCT. **(E)** Kruskal Wallis + Dunn's *Post-hoc* test. ^*^p < 0.05; ^**^p < 0.01; ^***^p < 0.001. Same groups as in Figure 1.

**Figure 9:**
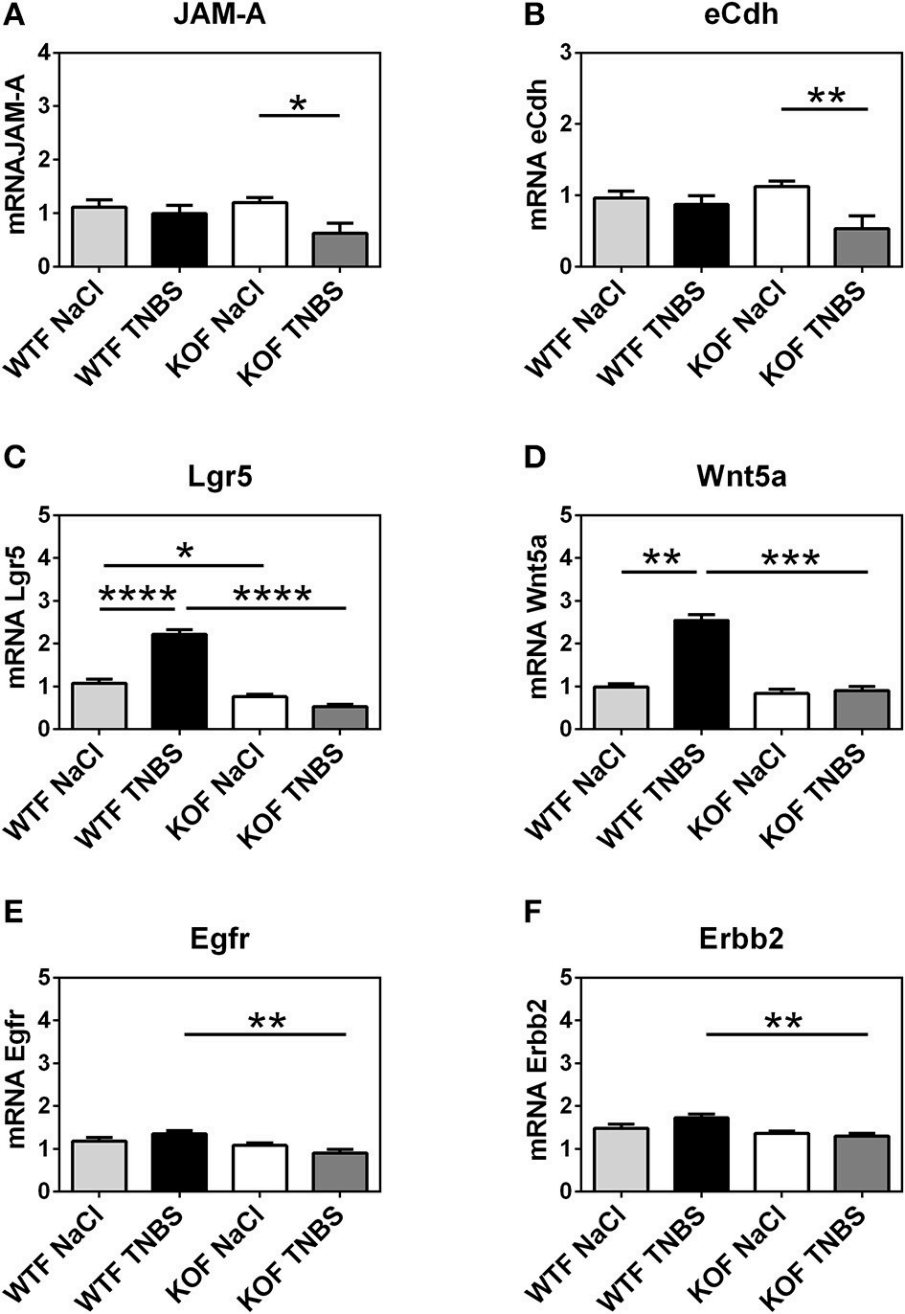
Effect of endogenous AM on epithelial cellular adhesions and regenerative responses during acute episode of colitis caused by rectal instillation of TNBS in females. Expression of 2 adhesion molecules and 4 regenerative biomarkers were measured by qRT-PCR in colonic tissue samples using GAPDH expression for normalizing mRNA levels. The absence of *adm* gene resulted in a significant decrease in the expression of JAM-A **(A)** and eCdh **(B)** in TNBS-treated KOF when compared with sham KOF and treated WTF. As it occurred in males, TNBS instillation did not cause any significant alteration in the expression of these molecules in the WTFs. Furthermore, TNBS caused a significant increase on the expression of LGR5 **(C)** and Wnt5a **(D)** in WTF that was not paralleled in KOF. Surprisingly, among basal conditions, sham KOF exhibit significant lower expression levels of Lgr5, a stem cell biomarker, than control WTF group. On the other hand, there was no increase on the levels of EGFR **(E)** and Erbb2 **(F)** on TNBS-treated WTF, but there was a significant decrease on the expression of these genes in TNBS-treated KOF when compared to their WT counterparts. **(A–E)** Data are shown as mean ± SEM. **(A,B,D)** Kruskal Wallis + Dunn's *Post-hoc* test. **(C,E,F)** Two way ANOVA + Tukey's MCT. ^*^p < 0.05; ^**^p < 0.01; ^***^p < 0.001; ^****^p < 0.0001. Same groups as in Figure 1.

Before analyzing qRT-PCR data, NormFinder software program was used for calculating the expression stability of several potential housekeeping genes in different sample sets. All reference genes were stable enough for all samples and GAPDH was used as the housekeeping gene (Figures 7–**9**).

To explore the potential mechanism of endogenous AM in protecting the colon we evaluated the expression levels of different cytokines (TNF-α, IL-1β and IL-6). No significant changes were observed in the gene expression patterns of these molecules (Kruskal Wallis; Dunn's *Post-hoc* test; *p* > 0.05; Figure 7).

### Endogenous adrenomedullin enhances the expression of some adhesion molecules in colonic epithelium

In an attempt to characterize the impact of AM on epithelial integrity we examined gene expression levels of eight different adhesion molecules (Table 1). Significant changes were only observed in the gene expression patterns of Junctional adhesion molecule-A (JAM-A) and e-cadherin (eCdh) (Figures 8, 9).

TNBS treatment did not cause any variation in the mRNA levels of JAM-A and eCdh among WT groups of either sex (Figures 8A,B, 9A,B; one way ANOVA, Tukey's MCT; *p* > 0.05). However, *adm* null-mice had a decreased expression of these two adhesion molecules. In KOM, the decrease is observed when they are compared with treated WTM: JAM-A (Figure 8A; one way ANOVA; Tukey's MCT; *p* < 0.05) and eCdh (Figure 8B; one way ANOVA; Tukey's MCT; *p* < 0.05). Meanwhile, among females, TNBS treatment decreased mRNA levels of these adhesion molecules in KOFs when compared with their sham group (Figure 9A; Kruskal Wallis; Dunn's *Post-hoc* test; *p* < 0.05 for JAM-A and eCdh) and with treated WTF (Figure 9A; Kruskal Wallis; Dunn's *Post-hoc* test; *p* < 0.05 for JAM-Aand eCdh).

### Adrenomedullin stimulates the expression of genes related with epithelial regeneration

We investigated the possibility of AM protecting colonic tissue against injury by promoting the expression of some genes related with colonic epithelial regeneration. Specifically we evaluated the expression of genes coding for Leucine-rich repeat-containing G-protein coupled receptor 5 (LGR5), Wnt5a, epidermal growth factor receptor (EGFR), and Erb-B2 Receptor Tyrosine Kinase 2 (Erbb2) (Table 1). LGR5 is a biomarker for adult stem cells in the colon (Beumer and Clevers, 2016). Wnt5a, a non-canonical Wnt ligand, seems to be required for crypt regeneration after injury in mice (Miyoshi et al., 2012). EGFR is a member of the ErbB family of receptors and it is essential for normal development of mammalian glands, furthermore, as a receptor for EGF, it stimulates epithelial cell growth and differentiation (Poyner et al., 2002). And, finally Erbb2, another receptor of the ErbB family which is also a receptor for EGF, is necessary for normal growth and epithelial cell mitosis (Riethdorf et al., 2008).

After TNBS treatment, expression of all these genes was enhanced in WTM when compared with their sham littermates (Figure 8C; one way ANOVA, Tukey's MCT; *p* < 0.001 for LGR5; Figure 8D; one way ANOVA, Tukey's MCT; *p* < 0.01 for Wnt5a; Figure 8E; one way ANOVA, Tukey's MCT; *p* < 0.01 for Egfr; Figure 8F; Kruskal Wallis; Dunn's *Post-hoc* test; *p* < 0.001 for Erbb2). Interestingly, TNBS-treated KOM did not show such an expression increase (Figure 8C; one way ANOVA, Tukey's MCT; *p* < 0.001 for Lgr5; Figure 8D; one way ANOVA, Tukey's MCT; *p* < 0.05 for Wnt5a; Figure 8E; one way ANOVA, Tukey's MCT; *p* < 0.01 for EGFR; Figure 8F; Kruskal Wallis; Dunn's *Post-hoc* test; *p* < 0.001 for Erbb2).

In WT females, TNBS administration caused a significant increment when compared with sham WTFs in LGR5 (Figure 9C; one way ANOVA; Tukey's MCT; *p* < 0.0001) and Wnt5a (Figure 9D; Kruskal Wallis; Dunn's *Post-hoc* test; *p* < 0.01). Again, this reaction was completely prevented by lack of AM (Figure 9C; one way ANOVA; Tukey's MCT; *p* < 0.0001 TNBS-WTF vs. TNBS-KOF for LGR5; Figure 9D; Kruskal Wallis; Dunn's *Post-hoc* test; *p* < 0.0001 TNBS-WTF vs. TNBS-KOF for Wnt5a). Surprisingly, under physiological conditions, untreated KOF exhibit a lower level of Lgr5 in their colons than WTF (*p* < 0.05; Figure 9C). Furthermore, the absence of endogenous AM resulted in a dampened expression of EGFR and Erbb2 in TNBS-treated KOF when compared with treated WTF, but there were no differences when compared with their untreated counterparts (Figure 9E; one way ANOVA; Tukey's MCT; *p* < 0.01 for EGFR; Figure 9F; one way ANOVA; Tukey's MCT; *p* < 0.01 for Erbb2).

## Discussion

In this study we have demonstrated that TNBS treatment in mice lacking the *adm* gene results in a more severe colitis episode, especially in females; confirming that AM, PAMP or the interaction of both molecules constitutes an intrinsic protective mechanism against intestinal pathology.

The choice of proper controls in experimental models involving KO animals is especially important. Among the several options, one of them was to compare mice with the same genotype (homozygous for the “floxed” allele, tetOCre, and rtTA) that were treated (KO) or not treated (WT) with doxycycline. However, we decided to treat both the KO (homozygous for the “floxed” allele, tetOCre, and rtTA) and the WT (homozygous for tetOCre, and rtTA; and with not “floxed” *adm* alleles) animals with the same dose of doxycycline for two main reasons. First, recent studies have shown that use of doxycycline modifies many cellular pathways in vertebrates (Larráyoz et al., 2012b), and second, as a potent antibiotic doxycycline may have an impact on microbiota composition and therefore in colitis development (Fava and Danese, 2011).

Laboratory studies have demonstrated beneficial effects of AM treatment for colitis symptoms in rodents (Talero et al., 2008). These results led to pilot studies of AM treatment in patients with refractory ulcerative colitis (Ashizuka et al., 2012, 2013b). The results strongly suggest that AM has potential as a new therapeutic agent for the treatment of IBD, thus intravenously administered AM reduced in all cases the disease severity index, accelerated healing of colonic injuries, and in several patients maintained clinical remission for over a year (Ashizuka et al., 2012). However, none of the published studies was able to explore the opposite situation, i.e. the influence of intrinsic AM on colitis. The scientific interest for investigating the impact of the lack of endogenous AM on colitis arises from the fact that IBD etiology, and especially the genetic component involved in the initiation of these pathologies, is still not completely understood. Cheung et al. (2011) described a single nucleotide polymorphism (SNP) close to the *adm* gene which causes a significant reduction in the plasmatic AM levels and correlated with cancer susceptibility (Martínez-Herrero and Martínez, 2013). Considering these two facts, it would be interesting to investigate if SNP-regulated endogenous AM levels are related with colitis initiation and progression, and whether this SNP is involved in the onset of IBD.

We chose the dose and volume of TNBS according to the methodology published by the group of Gonzalez-Rey et al. (2006). With these methods, our study has demonstrated that TNBS instillation causes mild colitis in WT animals, moderate colitis in KOM, and moderate to highly severe colitis in KOF. However, if we compare our results of TNBS instillation in WT animals with previous papers of TNBS-induced colitis (Gonzalez-Rey et al., 2006), we can see that our mice developed milder symptoms. This phenomenon could depend on two different factors. The first would relate to animals' strains; our mice belong to the C57BL/6J strain, while González-Rey et al. used Balb/c animals. Other studies have already shown that the strain used in colitis assays can influence the severity of the pathology developed by mice (Knod et al., 2014). The second factor could relate to the age and size of the mice. Usually, colitis studies are carried out on 8- to 10-week old mice. However, due to the gene recombination procedure and the subsequent 1 month rest period (to allow for restoration of the bacterial microbiota after treatment with doxycycline), the mice used in this study were 16-week old. Logically, being older, the organs of the mice are larger and the dose of TNBS administered in relation to colon weight and size was lower. This second hypothesis seems plausible if we consider that our severity results are similar to those obtained by the group of Kono et al. (2010) where they instilled a dose of 1.5 mg of TNBS (half of our dose) into 8-week old Balb/c mice. Nevertheless, the effects of TNBS on AM KO animals are clearly more severe than those observed on their WT littermates.

This study shows that lack of *adm* gene correlates with a faster initiation and an exacerbation of colitis symptoms, especially among females. Endogenous AM was able to prevent body weight loss, dehydration incidence, diarrhea, and rectal bleeding. Furthermore lack of AM and PAMP resulted in a significant increase in the weight/length ratio of the colon, which is in line with previous studies from our group (Martínez-Herrero et al., 2016a).

Data from macroscopic observation was further confirmed by microscopic analysis of colonic sections, reflecting an aggravation of mucosal disruption, edema, and inflammatory infiltration in KO mice. Instillation of TNBS caused a significant increase in the thickness of the colonic mucosa in both genotypes. These results are in line with previous results published by Kono et al. (2010). In TNBS-treated WT mice the histological structure of the tissue was maintained, and in treated WTF small and disperse foci of inflammatory cells were found. However, in absence of AM, TNBS treatment caused extensive areas of inflammatory cell infiltrates, and completely destroyed the microarchitecture of the colonic wall in some areas. In treated KO animals, but not in WT animals, a significant increase in the thickness of the submucosa could be also appreciated. Similar results had been previously described by the group of Ito et al. (2006) using a KO mouse model for the IFN-γ gene. In both genotypes, a more severe inflammation and tissue damage is again observed among females, confirming that they are more susceptible to TNBS-induced colonic damage. Therefore, endogenous AM protects the colon at a microscopic level during the acute colitis episode, decreasing lymphocyte infiltrates and preserving the microarchitecture of the tissue, thus maintaining its normal physiological functions.

It has been previously described that high amounts of Th1 cytokines (TNF-α, IL-1β, IL6; Talero et al., 2011) could be detected in colonic tissues of TNBS-treated animals. These are pro-inflammatory cytokines that, when uncontrolled, can lead to potent tissue injury. Our determination of local cytokine levels by qRT-PCR did not show any variation in TNBS-treated mice when compared with their respective controls at day five. This could be due to the fact that mRNA quantification was performed from the tissue obtained at the endpoint of the experiment, 5 days after instillation of TNBS. Probably the peak of cytokine synthesis occurs between 6 and 24 h after tissue damage (Carson et al., 2000; Chaluvadi et al., 2009), thus we may have lost the window of opportunity to measure these mediators.

In contrast with previous results, which postulated that the treatment with AM restored the expression of a plethora of tight and adherence junctions in colitis models (Ashizuka et al., 2009), we did not see any alteration on the levels of most of these molecules, except for the levels of expression of JAM-A and eCdh. The levels of these two adhesion molecules in the colon of treated null-mice are significantly lower, but no changes were observed in treated WTM. However, this result should be considered with caution. AM treatment increases normal AM circulating levels, whereas our WT animals had just normal levels of the peptide. This could mean that endogenous AM levels are sufficient to prevent the decline in the expression of some adhesion molecules, such as JAM-A and eCdh, but not enough to elicit the up-regulation of others, i.e., desmoglein2 or connexin-26.

Finally, we decided to analyze the expression levels of different genes related to the regeneration and renewal of colon epithelium, since we thought that AM could be exerting its protective role against colonic damage through this pathway. LGR5 is a biomarker for particular adult stem cells which act during homeostasis and are essential for regeneration (Beumer and Clevers, 2016). AM acts as a regulatory factor for other types of stem cells, such as neural stem cells (Martínez-Herrero et al., 2012), endothelial progenitors, or hematopoietic stem cells (Larráyoz et al., 2012b). In this work we have shown for the first time that AM also regulates gut LGR5-positive stem cells. TNBS treatment increased the expression of all studied regeneration genes in WTM when compared with control animals; meanwhile, in KOM the expression of these genes remained at the same level as those of their control littermates. In females, treated KOF showed a decrease of all the studied regeneration genes when compared with treated WTF. The results observed in the pattern of expression of these genes related with regeneration and renewal of the colon after damage support all the other results obtained during the experiments. WT animals exhibited mild symptoms of colitis, and this could be explained by the higher levels of expression of the four genes related with colonic epithelial regeneration.

An intriguing result of this study was that TNBS treatment seemed to have different effects between males and females. This phenomenon could be explained in part because of the significant differences of weight between males and females at the moment of TNBS instillation, which is in line with previously C57/BL6 phenotype characterization by The Jackson Laboratory^1^. In addition, sexual hormones may be also participating in these observed differences between sexes. However this is only a theory because the menstrual cycle stage of females had not been determined at the moment when the experiment began. Epidemiologic studies revealed a higher predisposition of women to suffer inflammatory intestinal pathologies, not only IBD (Harris et al., 2016; Fumery et al., 2017). In addition, the relation between etiology of IBD and the alteration of female sex hormones has already been demonstrated, since epidemiological data indicate that the consumption of oral contraceptives for long periods of time is considered a risk factor in the development of these pathologies (Ponder and Long, 2013). In fact, the strong connection between estrogen levels and the immune system has become evident from the study of postmenopausal osteoporosis (D'Amelio, 2013). Thus, a lower weight and a potentiation of the immune response mediated by estrogens could explain higher susceptibility of females to TNBS-induced colitis. However, treated KOF have been the more affected group in all the analyzed variables, suggesting that lack of AM potentiates the effects of sexual bias. This might be an additive effect of sex and *adm* deletion, but it could also be a real potentiation as far as AM is intimately related with female sexual hormones. Plasmatic AM levels increase during the follicular phase, and descend during the luteal phase of the estrous cycle (Marinoni et al., 2002). Furthermore, previous studies pointed out that AM may be an important regulator of progesterone production (Li et al., 2011). And it has also been shown that AM may have a paracrine effect on ovarian steroidogenesis (Li et al., 2008). Addition of AM inhibited FSH-induced estradiol secretion in 2-day-old follicles and also suppressed eCG- stimulated progesterone release in corpora lutea (Li et al., 2008). Finally, altered AM plasma levels have been found in women with pathologies associated to changes in normal sex hormones, such as osteoporosis (Lin et al., 2001) and primary dysmenorrhea (Dikensoy et al., 2008). In this scenario, although it is only a hypothesis, it seems plausible to argue that in KO females, apart from their lower weight, the absence of AM could cause an alteration in estrogen production or activity that would contribute to the development of a more serious immune deregulation than that observed in males.

In summary, in a model of acute colitis caused by rectal instillation of TNBS, endogenous AM is able to delay the development of IBD and prevent the onset of serious clinical signs such as anorexia, severe diarrhea, dehydration, and bleeding. Lack of AM leads to increased local inflammation at the macroscopic level, triggers an aberrant inflammatory response that causes infiltrations of numerous lymphocytes, especially at the level of the colonic mucosa, but also of the submucosa, which ends up destroying the typical crypt microarchitecture. The protective role of AM in the colon seems to be mediated by a control over the immune response, preservation of the integrity of the colorectal epithelium through the maintenance of adherence junctions, and the enhancement of the expression of genes related with regeneration and epithelial cell renewal. Finally, total lack of AM seems to have different effects in males than in females. In the case of colitis, AM absence causes more severe effects in females than in males, suggesting a more important protective role of this hormone in females.

## Author contributions

SM-H, IL, JN-Í, and SR-M performed experiments and interpreted data. SM-H and AM designed the study and wrote the manuscript. All authors read and approved the final version of the manuscript.

### Conflict of interest statement

The authors declare that the research was conducted in the absence of any commercial or financial relationships that could be construed as a potential conflict of interest.

## Footnotes

^1^Laboratory TJ. Mouse Phenome Database.

## Abbreviations

AM: adrenomedullin
CD: Crohn's disease
CLR: calcitonin receptor-like receptor
Ecdh: e-cadherin
EGFR: epidermal growth factor receptor
Erbb2: Erb-B2 receptor tyrosine kinase 2
H&E: hematoxylin and eosin
IBD: inflammatory bowel disease
IL: interleukin
JAM-A: junctional adhesion molecule-A
KO: knockout
Lgr5: Leucine-rich repeat-containing G-protein coupled receptor 5
MCT: multiple comparisons test
PAMP: proadrenomedullin N-terminal 20 peptide
qRT-PCR: quantitative real time-PCR
RAMP: receptor activity modifying protein
TLR4: toll-like receptor 4
TNBS: 2,4,6-trinitrobenzenesulfonic acid
TNF-α: tumor necrosis factor alpha
UC: ulcerative colitis
ZO-1: tight junction protein.
